# Influence of Time, Intensity, and Total Dose Parameters on the Ultrasound Effects of Percutaneous Electrolysis: An In Vitro Experimental Study

**DOI:** 10.3390/healthcare14040516

**Published:** 2026-02-18

**Authors:** Miguel Malo-Urriés, Jacobo Rodríguez-Sanz, Sergio Borrella-Andrés, Isabel Albarova-Corral, Erik Garcia-Ribell, José Antonio Gaitán-Villena, Carlos López-de-Celis

**Affiliations:** 1 Health Sciences Faculty, Department of Physiotry and Nursing, University of Zaragoza, 50009 Zaragoza, Spain; malom@unizar.es (M.M.-U.); sergiocai04@gmail.com (S.B.-A.); ialbarova@unizar.es (I.A.-C.); 2 PhysiUZerapy Health Sciences Research Group, University of Zaragoza, 50009 Zaragoza, Spain; 3 Department of Medicine, Faculty of Medicine and Health Sciences, Universitat Internacional de Catalunya, 08195 Sant Cugat del Vallès, Spain; erik.garcia@uic.es (E.G.-R.); kinegaitan@gmail.com (J.A.G.-V.); 4 ACTIUM Functional Anatomy Group, Faculty of Medicine and Health Sciences, Universitat Internacional de Catalunya, 08195 Sant Cugat del Vallès, Spain; carlesldc@uic.es; 5 Department of Physiotherapy, Faculty of Medicine and Health Sciences, Universitat Internacional de Catalunya, 08195 Sant Cugat del Vallès, Spain; 6 Study Group on Pathology of the Locomotor System in Primary Care (GEPALAP), Institut Universitari d’Investigació en Atenció Primària (IDIAP Jordi Gol), 08007 Barcelona, Spain

**Keywords:** percutaneous electrolysis, dosing, quantitative ultrasound, galvanic current, tendinopathy

## Abstract

**Highlights:**

**What are the main findings?**

- Current intensity is the main driver of electrochemical gasification of percutaneous electrolysis.
- Gas saturation occurs at specific intensity–time thresholds and compromises ultrasound visibility.

**What are the implications of the main findings?**

- Greater intensity and longer time generate a greater gas effect.
- High intensity/longer duration compromises visibility via gasification.

**Abstract:**

**Objective**: This study aimed to quantitatively analyze the influence of time, intensity, and total dose parameters on the electrolytic effect induced by percutaneous electrolysis on cadaveric patellar tendons and to determine the relationship between these parameters and the ultrasound-based quantitative response using the UZ_eDose tool. **Methods**: An in vitro experimental study was conducted on cadaveric patellar tendons. A total of 45 unique combinations of percutaneous electrolysis were applied, corresponding to 15 different application times (0 to 1200 s) and three intensities of galvanic current (0.1 mA, 1 mA, and 3 mA). The electrolytic effect was quantified immediately after each application using UZ_eDose. Additionally, ultrasound visibility was recorded as a binary variable (visible or non-visible). Descriptive graphical analysis, Spearman and point-biserial correlation tests, and multiple linear regression models were conducted to explore relationships between parameters and outcomes. **Results**: The intensity of current showed the strongest positive correlation with the UZ_eDose values (ρ = 0.606, *p* < 0.001), particularly when considering only cases with maintained ultrasound visibility. The total dose was also positively correlated with UZ_eDose (ρ = 0.486, *p* = 0.001), whereas time alone showed no significant correlation. Loss of ultrasound visibility was significantly associated with longer application times, higher intensities, and greater total doses (*p* < 0.001). Multiple linear regression models confirmed the predominant role of intensity in predicting the electrolytic effect, explaining up to 62.7% of the variance when excluding non-visible cases. **Conclusions**: The electrolytic effect, as quantified by UZ_eDose, is primarily influenced by the intensity of the current and the cumulative dose applied. However, excessive intensities and durations can lead to gas saturation, compromising ultrasound visibility. These findings suggest that both intensity and time should be carefully balanced to maximize the therapeutic effect while preserving imaging control, supporting the use of ultrasound-based quantification tools for optimized, individualized dosimetry.

## 1. Introduction

Therapeutic percutaneous electrolysis is a technique for the treatment of different musculoskeletal disorders [1], particularly noted for its ability to induce a controlled electrochemical response through the application of galvanic current via a dry needling procedure [2]. This procedure triggers a localized redox reaction, causing molecular decomposition of the target tissue and the release of hydrogen, sodium, and other gaseous by-products, which are detectable via ultrasound imaging [2,3,4,5,6,7].

In tendinopathies and soft tissue injuries, percutaneous electrolysis has demonstrated efficacy in modulating the inflammatory response, stimulating neocollagenesis, and accelerating tissue regeneration processes [2,3,4,5,6,7]. However, optimal application parameters (intensity, time, total charge) remain debated [2,3,4,5,6,7,8,9]. In clinical practice, this debate is often framed in terms of “low-dose” versus “high-dose” electrolysis strategies, with substantial variability in treatment algorithms depending on the device used and the individual practitioner. Current clinical protocols often rely on standardized configurations [10,11,12], overlooking dynamic interactions between intensity and time. Tendons are a primary focus due to frequent involvement in chronic degenerative conditions; studies suggest electrolysis aids inflammation modulation and healing, yet clinical evidence is limited and heterogeneous, particularly for structural and long-term functional outcomes [9,13]. This heterogeneity reflects not only biological variability but also differences in how electrolysis parameters are selected and monitored in daily practice. All electrolysis protocols aim to trigger regenerative responses in compromised connective tissue, especially in chronic tendinopathies with a disorganized collagen matrix [2].

Although some modern devices have incorporated dosing systems based on tissue resistance measurements, these approaches still do not consider the ultrasound response as an objective criterion for dosage adjustment [2,3,4,5,6,7,8,9]. Electrolysis induces identifiable ultrasound changes [7,14], such as the appearance of microbubbles and tissue gasification, which could serve as quantifiable technical markers of the electrochemical process to guide more precise dosage strategies [2].

There is a need to analyze how combined parameters, especially current intensity and application time, influence the ultrasound-detected effect of electrolysis. Furthermore, it is essential to determine thresholds beyond which the accumulated effect leads to gas saturation, compromising ultrasound visibility and limiting effective monitoring. In this context, ultrasound-detected gasification represents a technically measurable outcome that may help structure dosing strategies in a field currently characterized by wide clinical variability. While existing studies show various biological responses, they lack measurable data to guide clinical translation.

Recently, our team developed “UZ_eDose”, an AI-powered ultrasound image analysis software, which quantitatively analyzes ultrasound changes (especially those induced by percutaneous electrolysis) to provide objective data on tissue modifications [7,14]. Characterizing electrolysis-induced tissue variations via this objective, specific approach may enhance understanding and clinical interpretation of the technique’s effects.

Consequently, this study aims to quantitatively analyze the relationship between electrolysis parameters (time, intensity, total dose) and the ultrasound-detected effect on cadaveric tendon tissue using UZ_eDose for objective tissue gasification measurements. Additionally, it seeks to identify potential application thresholds, where ultrasound visibility becomes compromised, to establish safer, personalized dosing recommendations based on observed responses. It is important to note that the ultrasound-detected effects evaluated in this study primarily reflect electrochemical and physicochemical phenomena, particularly gas formation and tissue gasification induced by galvanic current application. While these changes may be indirectly related to biological responses described in previous studies, the present in vitro model does not allow direct assessment of cellular or regenerative processes. Therefore, the analysis focuses on the technical and quantitative characterization of the electrochemical effect observable by ultrasound.

## 2. Materials and Methods

### 2.1. Study Design

An in vitro experimental study was designed to analyze the ultrasound effect of different combinations of time, intensity, and total dose of percutaneous electrolysis on cadaveric tendon tissue. A total of 45 different combinations were applied, corresponding to 15 application times (ranging from 0 to 1200 s) and three galvanic current intensities (0.1 mA, 1 mA, and 3 mA), all delivered to cadaveric patellar tendons. The quantification of the galvanic effect was performed via ultrasound image analysis using the specific tool UZ eDose (Version: V.1.), which enables quantitative measurement of the degree of tissue electrolysis. Given the use of cadaveric tissue, the outcomes of this study are restricted to immediate electrochemical effects detectable by ultrasound and do not reflect biological responses, tissue regeneration, or cellular mechanisms.

This study was conducted within the framework of a project approved by the Local Ethics Committee of the Universitat Internacional de Catalunya (CBAS-2023-11). Ethical principles for the use of biological samples were respected, and good scientific practices were followed for tissue handling and assessment.

### 2.2. Sample

Forty-five human cadaveric patellar tendon samples were used. Samples were stored at −20 °C and acclimatized to room temperature for 48 h prior to the experimental procedures, ensuring standardized handling and application conditions.

### 2.3. Experimental Procedure

Each tendon sample received a randomly assigned electrolysis protocol corresponding to one of the 45 possible combinations between 15 application times (0, 1, 2, 3, 5, 10, 15, 30, 60, 120, 180, 300, 600, 900, and 1200 s) and 3 current intensities (0.1 mA, 1 mA, and 3 mA). Each combination was applied only once per sample. These protocols allowed a comprehensive evaluation of the effects of different temporal and dose configurations on tendon tissue. The total charge delivered in each application was calculated as the product of intensity (mA) and time (s), yielding 45 distinct dose values in millicoulombs (mC). Random assignment ensured procedural homogeneity and avoided systematic bias in the distribution of applied doses.

**Figure 1:**
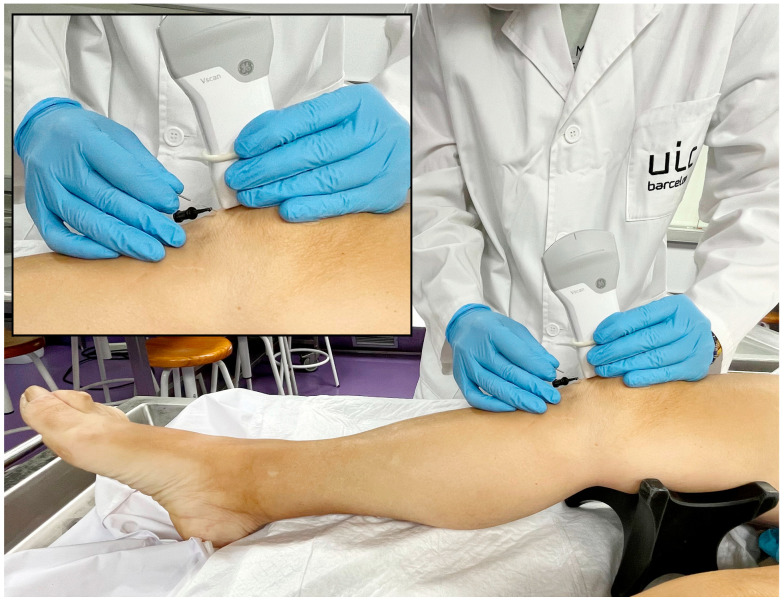
Performance of the ultrasound-guided percutaneous electrolysis technique and subject positioning.

The patellar tendon was placed in a standardized position, simulating the clinical posture for electrolysis treatment, with consistent 15° knee flexion across all samples (Figure 1). The procedure was ultrasound-guided, using a longitudinal in-plane approach with distal needle entry. The current application was performed by a specialist with over 10 years of experience in ultrasound-guided invasive procedures and specific training in percutaneous electrolysis. The needles used were 40 mm long and 0.30 mm in diameter (Agupunt), and the EPTE Bipolar System device was employed to ensure controlled delivery of galvanic current.

**Figure 2:**
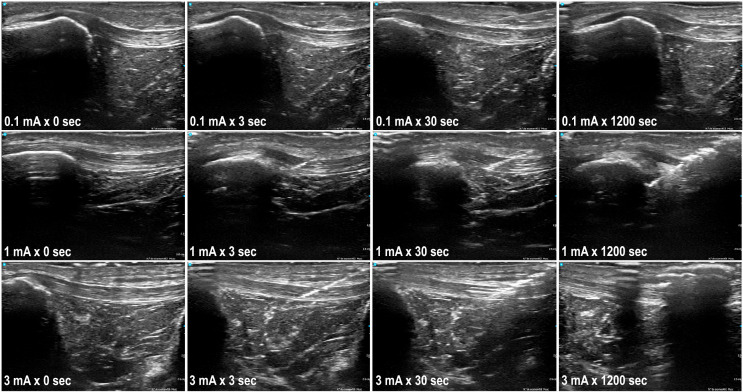
Gas effect of percutaneous electrolysis in ultrasound with different combinations of time and intensity.

Quantitative ultrasound measurements were performed immediately after each application using the UZ eDose tool. Standardized ultrasound evaluation was performed using a VScan Air device (General Electric, Cincinnati, OH, USA) with longitudinal in-plane imaging of the patellar tendon. Ultrasound acquisition settings were standardized across all measurements to ensure comparability between conditions. The same musculoskeletal preset was used for all samples, and all assessments were performed immediately after the current application under the same capture conditions. For each case, the best available image was selected to represent the treated region under consistent capture conditions. Measurements were conducted by an expert in musculoskeletal ultrasound with more than 15 years of experience, ensuring accurate and reproducible evaluation. The images were later processed with the UZ eDose tool, which is integrated within the UZ eDose tool software and specifically designed to objectively assess the degree of electrolysis [7,14]. The software provides quantitative metrics related to changes in tissue texture, echogenicity, and internal structure. Higher UZ eDose values reflect greater galvanic effect (e.g., presence of hydrogen and tissue gasification) and should be interpreted as a quantitative indicator of electrochemical gasification rather than of biological tissue response (Figure 2).

### 2.4. Statistical Analysis

Statistical analyses were conducted using IBM SPSS Statistics version 29.0 (IBM Corp., Armonk, NY, USA). Given the experimental design, where each time–intensity combination was applied only once and no replicates were included, a descriptive and correlational approach was used along with regression modeling to explore relationships between variables.

An initial exploratory graphical analysis was conducted to examine the behavior of the quantitative variable UZ_eDose as a function of application time and current intensity. Multiline plots were generated, with time (s) on the X-axis and UZ_eDose on the Y-axis. The three intensities (0.1 mA, 1 mA, and 3 mA) were plotted together for comparison. Similar graphs were also generated to explore the relationship between UZ_eDose and total charge (dose in mC), again distinguishing between intensities.

Spearman correlation analyses were conducted to examine the relationship between UZ_eDose, time, intensity, total charge, and ultrasound visibility. Pearson correlation coefficients range from −1 to +1, with absolute values increasingly close to 1 indicating an increasingly stronger relationship. Various cutoffs have been proposed to categorize the strength of the relationship using descriptors such as “weak” (r < 0.40), “moderate” (r = 0.40 to 0.69), or “strong” (r ≥ 0.70) [15,16]. Spearman correlation was chosen due to the non-normal distribution of the data and the ordinal nature of the binary visibility variable (0 = visible image, 1 = non-visible image). A significance threshold of *p* < 0.05 was used.

To determine the combined effect of the independent variables on ultrasound response, a multiple linear regression was performed using UZ_eDose as the dependent variable and time, intensity, and total dose as predictors. The model was evaluated using adjusted R^2^ and ANOVA for overall significance.

Due to the observation that certain time–intensity combinations generated excessive gas production, leading to loss of ultrasound visibility, an additional binary variable (“Visibility”) was introduced. Binary logistic regression was used to identify predictors of visibility loss, with time, intensity, and dose as explanatory variables. To control the potential bias introduced by visibility loss, a separate correlation analysis was performed using only the cases in which ultrasound visibility was preserved (visibility = 0). This allowed for more precise evaluation of the dose–response relationship in the absence of signal masking due to excessive gas.

Lastly, to assess whether different time–intensity combinations produced similar effects when the total charge was equivalent, comparisons of UZ_eDose values were conducted between equivalent-dose groups. Only samples with preserved visibility were included in this analysis. Non-parametric tests (Mann–Whitney U or Kruskal–Wallis) were used to identify significant differences between groups.

## 3. Results

### 3.1. Time-Intensity Response Patterns

**Figure 3:**
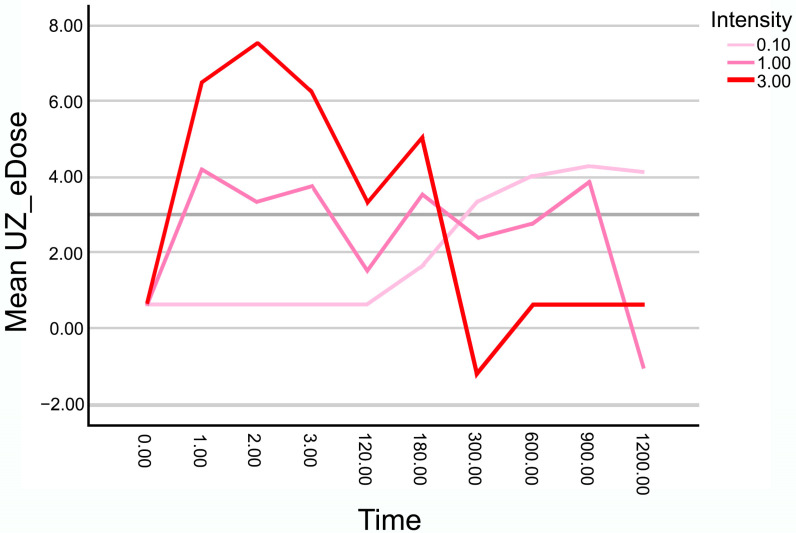
Temporal evolution of UZ_eDose as a function of application time for the three intensities used (0.1 mA, 1 mA, and 3 mA). Higher UZ_eDose means higher gas effect.

The evolution of the mean UZ_eDose as a function of application time for the three intensities used—0.1 mA, 1 mA, and 3 mA—is represented in Figure 3. The higher the mean UZ_eDose is, the greater the gas effect of the application. Apparent differences are observed in the behavior of each intensity over time.

For the lowest intensity (0.1 mA), the progression is more stable, with no abrupt decreases in UZ_eDose values. Levels remain low to moderate throughout the application time without reaching the saturation threshold observed at higher intensities.

In the case of 1 mA, the increase in UZ_eDose is more gradual, reaching a peak between 600 and 900 s, followed by a similar decline, also associated with excessive gas formation and partial loss of ultrasound visibility.

Finally, at the 3 mA intensity, a rapid and pronounced increase in the electrochemical gasification effect is noted, reaching peak values around 2–3 s. However, from approximately 180 s onward, the score drops sharply due to ultrasound visibility loss caused by tissue gas saturation, coinciding with the clinical observation of ultrasound visibility loss due to tissue gas saturation. Thereafter, values remain close to zero.

### 3.2. Dose-Intensity Response Patterns

**Figure 4:**
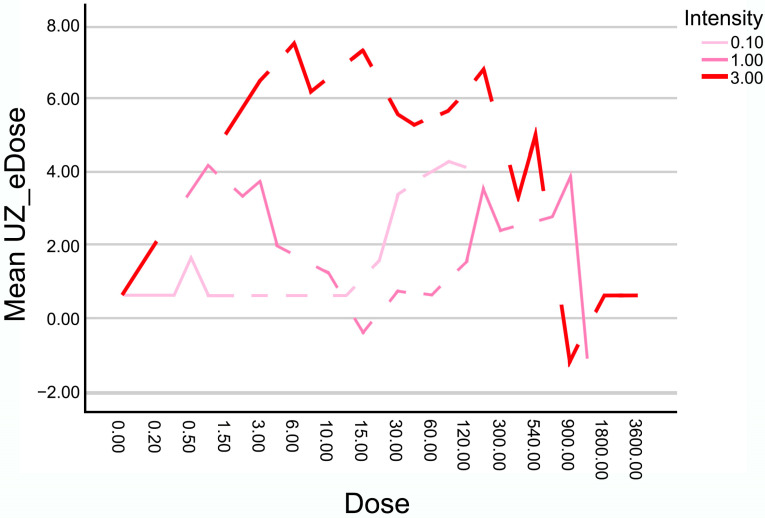
UZ_eDose progression as a function of total administered charge (mC) for the three intensities applied (0.1 mA, 1 mA, and 3 mA). Higher UZ_eDose means higher gas effect.

The evolution of the mean UZ_eDose as a function of the total applied dose (calculated as the product of current intensity and application time, in mC) for the three intensities analyzed (0.1 mA, 1 mA, and 3 mA) is shown in Figure 4. The results indicate that although some combinations reach similar total charge values, the ultrasound-detected gasification response measured by UZ_eDose is not uniform across intensities.

The lowest intensity (0.1 mA) yields low UZ_eDose values with a linear and gradual evolution without reaching saturation or interfering with visibility.

At 1 mA, the increase in UZ_eDose is more progressive, showing a sustained rise from 900 to 1200 mC without abrupt drops and maintaining high levels of measurable ultrasound-detected gasification.

In contrast, at 3 mA, UZ_eDose reaches high values from relatively low doses, peaking before 600 mC, and then dropping sharply once certain dose thresholds are exceeded. This decline corresponds to ultrasound visibility loss, likely due to excessive tissue gasification.

### 3.3. Correlation with Ultrasound Visibility

Correlation analysis revealed significant associations between the loss of ultrasound visibility and application parameters (time, intensity, and total dose) (Table 1).

Using the Spearman correlation coefficient, a significant negative correlation was observed between time and visibility (ρ = −0.475, *p* < 0.001), intensity and visibility (ρ = −0.346, *p* = 0.020), and total dose and visibility (ρ = −0.542, *p* < 0.001). To validate these findings, a point-biserial correlation analysis was also performed using Pearson’s coefficient, yielding consistent results: time showed a negative correlation (r = −0.597, *p* < 0.001), intensity (r = −0.360, *p* = 0.015), and total dose exhibited the strongest association (r = −0.852, *p* < 0.001). These results confirm that the loss of ultrasound visibility is influenced by both application time and current intensity, with a cumulative effect reflected in the total administered dose.

### 3.4. Correlation Analysis

Spearman correlation analysis revealed a significant positive correlation between the variable UZ_eDose and the applied intensity (ρ = 0.356, *p* = 0.016) (Table 2), indicating that the current intensity has a significant effect on the degree of ultrasound-detected electrochemical gasification change.

However, no significant correlation was found between time and UZ_eDose (ρ = −0.054, *p* = 0.725) nor between total dose and UZ_eDose (ρ = 0.133, *p* = 0.384).

Given that ultrasound visibility was lost in some high-intensity and long-duration applications, thus impairing accurate tissue quantification, additional Spearman correlation analysis was conducted, including only those cases in which ultrasound visibility was preserved. This approach aimed to evaluate the relationship between application parameters (time, intensity, and total dose) and the quantitative measure of ultrasound-detected electrochemical gasification without the confounding influence of excessive gas formation, which limits reliable assessment.

The Spearman correlation analysis performed exclusively on cases with preserved ultrasound visibility revealed a significant positive correlation between intensity and UZ_eDose (ρ = 0.606, *p* < 0.001) (Table 3). Similarly, a significant positive correlation was observed between total dose and UZ_eDose (ρ = 0.486, *p* = 0.001). No significant correlation was found between time and UZ_eDose (ρ = 0.178, *p* = 0.273). Excluding cases with ultrasound image loss revealed a direct relationship between both intensity and total dose with the degree of electrolysis observed, while time of application continued to show no significant correlation.

### 3.5. Multiple Regression Model on UZ_eDose

The results of the multiple linear regression model conducted on all data points, including both visible and non-visible ultrasound images, are in Table 4. The analysis shows the independent contribution of application time, intensity, and total dose to the variation in UZ_eDose. Current intensity (mA) was the most influential predictor of the ultrasound-detected gasification effect quantified by UZ_eDose, with a standardized coefficient of β = 0.801 (*p* < 0.001). Application time (s) also showed a significant positive effect (β = 0.504, *p* = 0.003), although to a lesser extent. In contrast, total dose (mC) was negatively associated with UZ_eDose (β = −0.906, *p* < 0.001), suggesting that the accumulated charge alone does not determine the ultrasound-detected gasification outcome, but rather how it is distributed over time and intensity. The overall model was statistically significant (F = 15.164, *p* < 0.001), explaining 52.6% of the variance (R^2^ = 0.526; adjusted R^2^ = 0.491).

Given that ultrasound visibility was lost in certain combinations involving higher intensities and longer durations, a second model was conducted, excluding these cases (Table 5). This filtered analysis showed improved explanatory power (adjusted R^2^ = 0.627), confirming intensity as the strongest predictor (β = 0.879, *p* < 0.001), followed by time (β = 0.521, *p* < 0.001). At the same time, total dose no longer reached statistical significance (β = −0.232, *p* = 0.073). Compared to the model including all cases, this filtered model demonstrated greater explanatory power, providing a more accurate representation of the relationship between application parameters and the quantified ultrasound response before visibility loss affected the measurement. The overall model was statistically significant (F = 22.889, *p* < 0.001).

## 4. Discussion

The primary aim of the present study was to evaluate the combined effect of application time, current intensity, and total administered charge during percutaneous electrolysis on the tissue response as quantified by ultrasound, as well as to identify potential saturation thresholds that impair ultrasound visibility of the treated tissue. Our results demonstrated that current intensity is the most influential factor in determining the magnitude of the ultrasound-detected effect (as measured by UZ_eDose), followed by application time. However, total charge alone did not fully explain the observed response, emphasizing the importance of how the charge is delivered through different intensity–time combinations. We also identified that beyond specific thresholds of intensity and duration, the electrolytic process induces excessive tissue gasification, which interferes with ultrasound visibility and limits the ability to monitor the procedure in real-time. The combination of higher intensity and prolonged duration produces a cumulative electrolytic effect, which may eventually lead to excessive gas formation and loss of ultrasound visibility. In contrast, lower intensities seem to allow for longer application times without compromising image quality. These findings provide valuable insight for optimizing dosage protocols in percutaneous electrolysis, emphasizing the importance of balancing intensity and application time.

Intratissue percutaneous electrolysis involves the application of a galvanic current through a needle inserted into the affected tissue, triggering electrochemical reactions that, in vivo, have been associated with physiological responses related to tissue repair [2]. The electrical current induces an electrochemical reaction that generates a controlled inflammatory response, activating cellular mechanisms responsible for phagocytosis and tissue repair [2,3,4,5,6,7]. At the chemical level, the process of electrolysis leads to the dissociation of water molecules (H_2_O) into hydrogen gas (H_2_) and hydroxide ions (OH^−^) at the cathode and the generation of oxygen gas (O_2_) and protons (H^+^) at the anode [17]. These reactions contribute to a localized pH shift and the formation of gas microbubbles within the tissue, which are detectable via ultrasound imaging and serve as a visual indicator of the electrolytic effect [7]. However, there is still insufficient research analyzing how each parameter—such as current intensity, application time, and total delivered dose—contributes to the magnitude and characteristics of the electrolytic effect within the tissue [7].

Current intensity consistently emerged as the most influential variable for ultrasound-observed electrolytic effect across the entire dataset and cases with preserved visibility. The significant positive association between intensity and UZ_eDose highlights its pivotal role in modulating tissue response. These findings support physiological evidence that higher intensities induce more pronounced electrochemical reactions, leading to enhanced gas production and greater ultrasound-detectable electrochemical changes. In vivo studies have associated higher intensities with inflammatory modulation [2,3,4,5,6,7]. Importantly, this influence remained robust even after accounting for potential confounders like gas saturation affecting image quality. Earlier studies, for example, show that low-intensity galvanic current produces local analgesia, while, in in vivo studies, higher intensities have been associated with tissue repair mediated by controlled inflammatory responses [9].

Application time’s role was more nuanced. In the full dataset, time moderately contributed to the electrolytic response, less so than intensity. However, when analyzing cases with preserved ultrasound visibility, time showed a positive relationship and became a significant predictor in the multiple regression model. This suggests time meaningfully contributes to the overall effect when ultrasound assessment is maintained, though its impact may be obscured by excessive gasification. This interaction between intensity and time supports the interpretation of electrolysis as a nonlinear phenomenon, where similar total charges delivered through different intensity-time combinations result in markedly different electrochemical dynamics and ultrasound-detected effects. Thus, time alone may not drive the response but facilitate it, especially with higher intensities. We cannot attribute gas changes solely to biological effects. An example is the study by Peñin-Franch [8], where greater effects on IL1B and LDH were observed when applying 3 mA for 48 s compared to 6 mA for 34 s. Both applications delivered the same electrical charge (144 mC), which supports the hypothesis that identical charges delivered with different intensities and durations do not produce the same effects. Moreover, these results suggest that, in terms of biological effects, application time might be more relevant than intensity in certain situations.

Total electrical dose (intensity × time) did not independently explain tissue electrolysis in the full dataset; no significant correlations or a negative association with UZ_eDose were observed in the regression model. However, excluding cases with visibility loss revealed a moderate positive correlation with quantified tissue response, though total dose did not reach statistical significance in the filtered regression model. These findings suggest that total charge alone is an insufficient predictor of electrolytic effect; its delivery mode, particularly through intensity, plays a more critical role in shaping tissue outcomes.

A key finding was identifying a threshold beyond which intense electrolytic application compromises ultrasound visibility. This image quality loss was evident in high-intensity, long-duration applications due to excessive gas production interfering with tissue assessment. Importantly, visibility loss was generally progressive, characterized by a gradual accumulation of hyperechoic gas artifacts that increasingly masked tendon echotexture until a saturation threshold was reached, after which the ultrasound image became non-interpretable. Specifically, visibility loss occurred with 3 mA for 180 s and 1 mA for 900 s, indicating a saturation point where ultrasound imaging becomes unreliable. This intensity-dependent threshold emerges earlier at higher current levels, reinforcing that both total accumulated dose and its delivery (time × intensity) are crucial. From a mechanistic perspective, this behavior is consistent with a saturation-driven electrochemical–mechanical model, in which excessive gas accumulation acts as a physical masking factor, limiting ultrasound signal transmission rather than reflecting proportional increases in electrochemical activity. Incorporating a binary visibility variable allowed objective quantification; analyses confirmed that time, intensity, and total dose were significantly associated with image loss. This highlights that the electrolytic dose cannot be increased indefinitely without compromising procedural monitoring. Avoiding ultrasound quantification beyond saturation prevents misinterpretation, ensuring treatment effects are assessed under reliable visibility. Clinically, this emphasizes defining maximum application times per intensity and personalizing parameters based on patient/injury specifics for effectiveness and safety. These ultrasound-detected gasification patterns are not exclusive to experimental models. Clinical case series in patients with patellar tendinopathy have reported similar ultrasound-visible microbubble formation and intensity-dependent gas accumulation during percutaneous electrolysis, concomitant with improvements in pain and function [18]. While such clinical observations cannot be directly extrapolated from the present in vitro findings, they underscore the relevance of understanding gas-related ultrasound phenomena for optimizing procedural monitoring and parameter selection in clinical practice.

This study has several limitations. First, as an in vitro cadaveric tendon model, physiological conditions differ from living tissue, as the absence of metabolism, inflammatory response, and cellular activity precludes direct assessment of biological processes such as tissue repair or regeneration. Consequently, the observed ultrasound-detected changes must be interpreted as physicochemical manifestations of electrolysis rather than biological responses. In addition, only one tendon type (patellar tendon) was analyzed, which may limit the extrapolation of these findings to tendons with different collagen architecture, composition, and mechanical properties, such as the Achilles or supraspinatus tendons. Second, the lack of replicates for each time/intensity combination limits robust inferential analyses and generalization; each combination was applied once, preventing within-condition variability estimation and restricting analysis to a descriptive, exploratory approach. This design was intentionally conceived to map a wide parameter space and identify global response patterns and saturation phenomena rather than to establish definitive dose–response relationships. Third, ultrasound measurements were obtained only immediately after electrolysis, without longitudinal follow-up, preventing evaluation of the medium- to long-term evolution of the electrochemical effect. Additionally, excessive gas formation in some high-intensity, prolonged-application combinations resulted in ultrasound visibility loss, impairing quantitative assessment despite the use of a binary visibility variable to account for this limitation. Furthermore, no histological or microscopic confirmation was performed, limiting interpretation of gasification phenomena to ultrasound-detected changes and precluding direct correlation with underlying tissue microstructural or cellular alterations. Beyond intervention-specific parameters, musculoskeletal tissue behavior and symptom expression are influenced by multiple external and contextual factors, including environmental variables, which have been shown to modulate pain perception and tissue irritability over time [19]. In this context, objective ultrasound-based quantification tools may play a key role in reducing interpretative variability and improving the consistency of electrolysis parameter selection across heterogeneous clinical scenarios. Finally, statistical analysis focused on correlations and linear regression; more complex models (nonlinear, interaction terms) were not explored but could explain additional variance. Future studies should incorporate experimental replication, in vivo tissue models, longitudinal assessment, histological validation, and advanced statistical approaches to better integrate technical findings with biological interpretation.

## 5. Conclusions

This experimental study explored the relationship between electrolysis parameters (application time, current intensity, and total electrical dose) and ultrasound-detected gas formation on cadaveric tendon tissue. The findings indicate that current intensity is the primary determinant of tissue gasification quantified by UZ_eDose, with application time acting as a relevant modulatory factor. Conversely, total electrical dose alone inconsistently explained tissue response, suggesting that the delivery mode, particularly the time and intensity combination, may be more relevant than accumulated charge.

Additionally, the study identified a potential saturation threshold where excessive gasification limits ultrasound visibility, especially in high-intensity, long-duration applications. This phenomenon may limit real-time monitoring and quantitative assessment of the electrolytic effect.

These results describe the electrochemical and ultrasound-detectable effects of percutaneous electrolysis under controlled in vitro conditions and should not be interpreted as direct evidence of biological tissue response. Nevertheless, the findings highlight clinically relevant technical considerations, including parameter selection and ultrasound visibility constraints, that may contribute to safer, more consistent, and better-monitored electrolysis protocols. Further in vivo research is necessary to validate the findings and inform safer, more effective, personalized percutaneous electrolysis approaches.

## Figures and Tables

**Table 1:** Correlation between ultrasound visibility and application parameters using Spearman and Pearson (point-biserial) coefficients.

Variable	Spearman Correlation	Spearman *p*-Value	Pearson Coefficient (r)	Pearson *p*-Value
Time vs. Visibility	−0.475	<0.001	−0.597	<0.001
Intensity vs. Visibility	−0.346	0.020	−0.360	0.015
Total Dose vs. Visibility	−0.542	<0.001	−0.852	<0.001

**Table 2:** Spearman correlation between UZ_eDose and application parameters.

Variable	Spearman Correlation	*p*-Value
Time vs. UZ_eDose	−0.054	0.725
Intensity vs. UZ_eDose	0.356	0.016
Total Dose vs. UZ_eDose	0.133	0.348

**Table 3:** Spearman correlation between UZ_eDose and application parameters, considering only cases with preserved ultrasound visibility.

Variable	Spearman Correlation	*p*-Value
Time vs. UZ_eDose	0.178	0.273
Intensity vs. UZ_eDose	0.606	<0.001
Total Dose vs. UZ_eDose	0.486	0.001

**Table 4:** Multiple linear regression model predicting UZ_eDose based on application time, current intensity, and total dose (all cases included).

Variable	B (Coef.)	Std. Error	Standardized Beta	t	*p*-Value	Adjusted R^2^	F	*p*-Value (Model)
Constant	633	445	-	1.423	162	0.491	15.164	<0.001
Time (s)	3	1	504	3.112	3			
Intensity (mA)	1.534	243	801	6.301	1			
Dose (mC)	−3	1	−906	−5.159	1			

Abbreviations: s, seconds; mA, milliampere; mC, millicoulomb.

**Table 5:** Multiple linear regression model predicting UZ_eDose based on application time, current intensity, and total dose (only cases with preserved ultrasound visibility).

Variable	B (Coef.)	Std. Error	Standardized Beta	t	*p*-Value	Adjusted R^2^	F	*p*-Value (Model)
Constant	0.469	0.369	-	1.270	0.212	0.491	15.164	<0.001
Time (s)	0.004	0.001	0.521	4.053	<0.001			
Intensity (mA)	1.687	0.210	0.879	8.019	<0.001			
Dose (mC)	−0.003	0.001	−0.232	−1.844	0.073			

Abbreviations: s, seconds; mA, milliampere; mC, millicoulomb.

## Data Availability

The raw data supporting the conclusions of this article will be made available by the authors on request.
